# Transpathology: molecular imaging-based pathology

**DOI:** 10.1007/s00259-021-05234-1

**Published:** 2021-02-13

**Authors:** Mei Tian, Xuexin He, Chentao Jin, Xiao He, Shuang Wu, Rui Zhou, Xiaohui Zhang, Kai Zhang, Weizhong Gu, Jing Wang, Hong Zhang

**Affiliations:** 1 Department of Nuclear Medicine and PET Center, The Second Affiliated Hospital of Zhejiang University School of Medicine, 88 Jiefang Road, Hangzhou, 310009 Zhejiang China; 2 Institute of Nuclear Medicine and Molecular Imaging of Zhejiang University, Hangzhou, China; 3 Key Laboratory of Medical Molecular Imaging of Zhejiang Province, Hangzhou, China; 4 Department of Medical Oncology, The Second Affiliated Hospital of Zhejiang University School of Medicine, Hangzhou, China; 5 Laboratory for Pathophysiological and Health Science, RIKEN Center for Biosystems Dynamics Research, Kobe, Hyogo Japan; 6 Department of Pathology, Children’s Hospital, Zhejiang University School of Medicine, Hangzhou, China; 7 College of Biomedical Engineering & Instrument Science, Zhejiang University, Hangzhou, China; 8 Key Laboratory for Biomedical Engineering of Ministry of Education, Zhejiang University, Hangzhou, China

**Keywords:** Molecular imaging, Pathology, Transpathology, Digital pathology

## Abstract

Pathology is the medical specialty concerned with the study of the disease nature and causes, playing a key role in bridging basic researches and clinical medicine. In the course of development, pathology has significantly expanded our understanding of disease, and exerted enormous impact on the management of patients. However, challenges facing pathology, the inherent invasiveness of pathological practice and the persistent concerns on the sample representativeness, constitute its limitations. Molecular imaging is a noninvasive technique to visualize, characterize, and measure biological processes at the molecular level in living subjects. With the continuous development of equipment and probes, molecular imaging has enabled an increasingly precise evaluation of pathophysiological changes. A new pathophysiology visualization system based on molecular imaging is forming and shows the great potential to reform the pathological practice. Several improvements in “trans-,” including trans-scale, transparency, and translation, would be driven by this new kind of pathological practice. Pathological changes could be evaluated in a trans-scale imaging mode; tissues could be transparentized to better present the underlying pathophysiological information; and the translational processes of basic research to the clinical practice would be better facilitated. Thus, transpathology would greatly facilitate in deciphering the pathophysiological events in a multiscale perspective, and supporting the precision medicine in the future.

## Introduction

Pathology is a branch of medical science, aiming at studying the cause of disease, exploring the pathophysiological processes, and providing necessary theoretical basis for the prevention and treatment of diseases [1]. The field of pathology has undergone revolutionary development in recent years. With the emergence of a large amount of new advanced technologies, including fluorescence in situ hybridization, next-generation sequencing, and spatial transcriptomics, the pathological analysis has stepped to the molecular level [2, 3]. Besides, the display mode of specimens is getting digital with the continuous spread of whole-slide imaging technology, resulting in increasing applications of artificial intelligence (AI) and label-free slides imaging technologies [4–6]. However, challenges facing pathology, such as the persistent concerns on the sample representativeness caused by the intrinsic heterogeneity in lesions, and the potential damage in pathological practice, still limit the development and wider use of pathology in clinical medicine.

Accompanied with the dramatic evolution of biological and instrument science, medical imaging technologies have greatly advanced over the past couple of decades. Overall information of individuals, including structural, functional, and molecular changes, could be obtained via multiple imaging methods [7]. Especially, with the development of molecular imaging, biological processes at the molecular level now can be visualized, characterized, and measured in living subjects [8]. Continuous innovation of imaging equipment and probes has further improved the ability of molecular imaging to noninvasively evaluate pathophysiological alternations, making the diagnostic capabilities increasingly approach the level of pathological practice [9, 10].

The transformation of either pathology or imaging is blurring the boundary between these two disciplines and leads to a trend of integration, which may bring forth a new pattern of pathological practice to better decipher the pathophysiological events in a multiscale perspective in vivo [11]. This article will briefly review the development of pathological practice, address the ongoing changes of pathology during the integration with molecular imaging, and discuss the prospects for the new pattern of pathological practice.

## The development of pathology

Historically, pathology first emerged in the Renaissance, mostly focusing on macroscopic or gross analysis of anatomy. In this period, increasing autopsy formed the basis for understanding normal anatomy, and accumulating evidence of abnormal structure observed led to the development of morbid anatomy [12]. It was soon realized that structural alteration can be used to explain the causes of disease and death. In the nineteenth century, the practice of pathology then underwent a revolutionary change with the development of a light microscope as well as the cell theory made by Virchow [13]. The theory of cellular pathology emphasized that disease arose primarily in individual cells, leading to the integration of abnormal cellular patterns in understanding various diseases. Subsequently, with the discovery of antibodies and antibody-antigen binding reactions, the immunohistochemistry (IHC) technique, which enabled the detection of tissue antigens with specially labeled antibodies that can be visualized at the light microscopic level, was developed in the 1940s. IHC was further promoted by the advent of monoclonal antibodies three decades later, which drives pathology to a more precise and complex level [14]. As the immunohistochemistry enabled pathologists to localize and quantify the protein expression, researchers and clinicians then could explore the pathogenic mechanism in a more basic perspective. In recent years, with the invention of technologies more advanced like polymerase chain reaction, fluorescence in situ hybridization, and next-generation sequencing, the field of pathology has been taken down to analysis of genetic level [15]. Pathology is constantly developing towards microcosm, and the combination of analytic techniques at different levels would provide multiscale pathophysiological information.

Advances in technology are now driving another major shift of current pathological paradigm towards digital. The practice of capturing static images of tissue sections or live stream a microscopic image using cameras was named digital pathology [16]. Recently, based on the development of modern computer and electronic systems, especially the advent of whole-slide imaging (WSI) technology, which enabled digitization of histological slides, the field of digital pathology has greatly evolved [17]. The remote pathology analysis of histological slides using digital image transmission, namely telepathology, is getting increasingly used in routine practice [18, 19], demonstrating a great potential to solve the problem of uneven distribution of medical resources. What’s more, traditional glass slides are fragile and hard to preserve for long, and the discoloration of tissue slides is inevitable [20], even for some rare and precious histological sections. Thus, the advantages of digital pathology, which can be stored with high-resolution and constant quality, and enabling the images to be accessed from any location with an Internet connection [21], greatly raise the effectiveness of pathology training, as well as communication between different centers.

Following the large-scaled digitalization of pathological sections, computerized image analysis methods have also garnered significant interest [22]. By using those informatics and big data analytic methods, the patterns of images that are beyond human perception, including texture features and much subcellular information, now could be identified and extracted automatically. In a recent study, an automated system was used to quantify histological changes in the hematoxylin-eosin (H&E) staining slides in an inflammatory bowel disease model, and 88% of the scores were consistent with pathologists [23]. And another automated image analysis algorithm was able to quantify colon-infiltrating macrophages, neutrophils, B cells, and T cells in immunohistochemical stained sections [23]. These techniques greatly reduced the workload of searching interested regions and improved the inter-/intra-observer consistency for pathologists. More recently, advanced label-free slide imaging methods have been developed. Tumor now can be delineated in nonneoplastic tissue based on the different Raman spectra derived from stimulated Raman scattering microscopy [6]. And features acquired from annotation-free histopathology images can be utilized to predict cancer recurrence with higher accuracy than pathologists by using deep learning algorithms [5]. Those label-free techniques provided more sophisticated chemical information and eliminated the need for stains and, meanwhile, relied more on computerized methods.

In the course of pathology development, as a bridge between basic researches and clinical medicine, pathology has significantly expanded our understanding of the diseases and exerted enormous impact on the management of patients [24]. Technological advances are continuously propelling the field of pathology forward; however, some drawbacks facing pathology deserve attention. To begin with, obtaining the pathological tissue through biopsy or operation is an invasive process, which may cause various complications such as excessive bleeding, infection, and tissue injury, especially in some vulnerable organs like the brain and lung [25, 26]. And in patients with malignancies, the invasive biopsy procedure may lead to needle tract seeding and metastases [27]. The potential damage mentioned above would inevitably affect patients’ acceptance to invasive procedures, particularly in some longitudinal studies that require repeated sampling [28, 29]. Since the limited specimen could not always be fully representative of the entire pathological tissue, especially in tumor lesion which is highly heterogeneous [30], great attention should be taken on the accuracy of tissue sampling. Besides, the inter- even intra- observer difference of diagnosing could be observed because of the inherent heterogeneity over the tissue section [31]. Furthermore, due to the complex preparation steps including fixation, dehydration, embedding, and staining, it always takes substantial time to make the final diagnosis [32]. What’s more, the specimen provides only static pathophysiological information of tissue, making it hard to map the disease dynamics. Lastly, though the digital pathology improved the processes of slides displaying and information transmission, drawbacks more vital, the invasiveness during biopsy or operation, and the concerns about sampling accuracy or interobserver variation caused by heterogeneity, still limit the development of pathology [33].

## Integration of pathology with molecular imaging

The development of molecular pathology and digital pathology is undoubtedly towards a pattern more representative of the pathological changes, presenting the lesion in a three-dimensional mode with less image interruption [33, 34]. Currently, the medicine is undergoing a tremendous transformation to precision medicine, and a trend of integration between the pathology and molecular imaging is emerging, which is conceived to transform the field of pathology. Major changes during this transformation reflect in the following regards.

### Real-time visualization of pathophysiological processes

Pathology would noninvasively monitor the in vivo dynamic changes of the physiological and pathological processes spatiotemporally. A recent study reported that radiologic imaging markers were correlated with expressions of molecular and genetic biomarkers [35]. Imaging biomarkers are particularly attractive, as they can be used in a noninvasive (or mildly invasive) manner, to evaluate the pathophysiological processes in the course of disease onset and progression [36]. In particular, imaging biomarkers have the potential to reveal the pathological changes early after treatment, thus providing an opportunity to tailor treatment based on the observed response [37, 38]. Noninvasive detection of molecular markers by molecular imaging techniques, such as positron emission tomography (PET), can allow for a much earlier disease diagnosis and evaluation than structure imaging methods, further improving staging and management [39–41]. Besides, it is reported that the ability of PET for tumor detection and evaluation would be further developed when multiple tracers were utilized simultaneously [42, 43]. However, it is worth noting that each of the existing imaging methods has its limitations, which promoted the development of hybrid imaging techniques, including the physical combination of PET and computed tomography (PET/CT), or PET and magnetic resonance imaging (PET/MRI) [44]. Hybrid imaging provides a wealth of information encompassing anatomical, functional, and molecular data, which could give a whole-body readout in an intact system and aid in lesion detection, patient stratification, and individualized treatment [45–47].

### Overall assessment of the whole-body pathophysiological state

Pathology would provide a high-precision depiction and full understanding of disease phenotype at the whole-body level, leading to the most appropriate therapeutic strategy depending on the stage and biological features of the disease [48]. Traditional pathology attempts to identify single molecular or histomorphological features that could be utilized for prognosis or prediction of response to drug therapy. However, disease evolution includes very complex processes with multiple molecular interactions in the cell and the microenvironment, no single biomarker can be used to characterize disease comprehensively [49]. Advances in the genomics, proteomics, transcriptomics, epigenomics, and the emerging field of image analysis-based phenomics are now able to add valuable information to the systemic understanding of complex disease mechanisms [50]. Integrating large-scale data from multi-omics fields may help to create a better understanding of the multiple molecular interactions occurring within the cell [51]. In this scope, the future development trend of pathology would be combining large and complex data sources from various omics fields that are reflective of histopathology, morphometrics, and spatial heterogeneity. With the fusion of the morphological and molecular signatures and clinical characteristics to form a large amount of high-quality multiscale data, future pathology would become more comprehensive and accurate, driving traditional diagnostic medicine from broad population-based prediction to a more personalized and precision-based science.

### AI in digital pathology and imaging

The pathology after integration with molecular imaging would overcome the sampling bias and inherent cognitive and visual traps due to the intralesional heterogeneity and the pathologists’ subjective decisions, so as to conduct a detailed and generous evaluation of the entire lesion. Over the last decade, the advent of whole-slide imaging and digital pathology has led to the advancement of computer-aided examination of tissue via digital image analysis, for example, AI [22, 52]. AI approaches enable the possibility of mining “sub-visual” image features from digital pathology slide images, and offer the opportunity to better model the disease quantitatively [22]. Moreover, digital pathology could serve as a bridge between pathology and radiology. It is clear that molecular changes in gene expression could generate a structural change in phenotype which is in turn observable on the imaging modality. For instance, tumor morphology in standard H&E tissue specimens reflects the functional status of the cells [53]. Likewise, radiographic imaging modalities such as MRI and PET are ultimately capturing structural and functional attributes which reflect the cellular morphology and pathophysiological processes characterizing the disease. The concept of radiology-pathology fusion has been around historically [54]. Recent studies emphasized the importance of aligning in vivo radiographic imaging and ex vivo histology in order to spatially map the extent of pathology onto the corresponding imaging [55]. This radiology-pathology co-registration could enable studies connecting spatially resolved genomics data with imaging biomarker, and hence better disease characterization.

## The concept of “transpathology”

With the advent of the post-genome era, the emergence of high-throughput and high-sensitivity analysis technologies, the combination of information and AI technologies, and the penetration of various emerging technologies into various fields of medicine, medicine has entered a period of rapid development. The opportunity to further develop for pathology now resides in integrating with a noninvasive, safe, and fast examination method which can match the traditional biopsy diagnosis value. Molecular imaging is the most promising field showing the potential to objectively evaluate pathological changes and achieve the integration. This integration would result in a new pattern of pathological practice. We hereby propose, for the first time, the concept of “transpathology” to describe this new type of pathological practice, whereby pathological lesions could be visualized in a trans-scaled mode using molecular imaging-based technologies. Transpathology holds the great potential to transparentize tissue and better present the underlying pathophysiological information, as well as to better facilitate the translational processes from the bench to the bedside. Transpathology can finely display a variety of biological features of the lesions at multiscale levels, and the image results obtained are directly presented in a digital mode, which is also convenient for storage and analysis.

## The molecular imaging-based multiscale pathology visualization

**Fig. 1:**
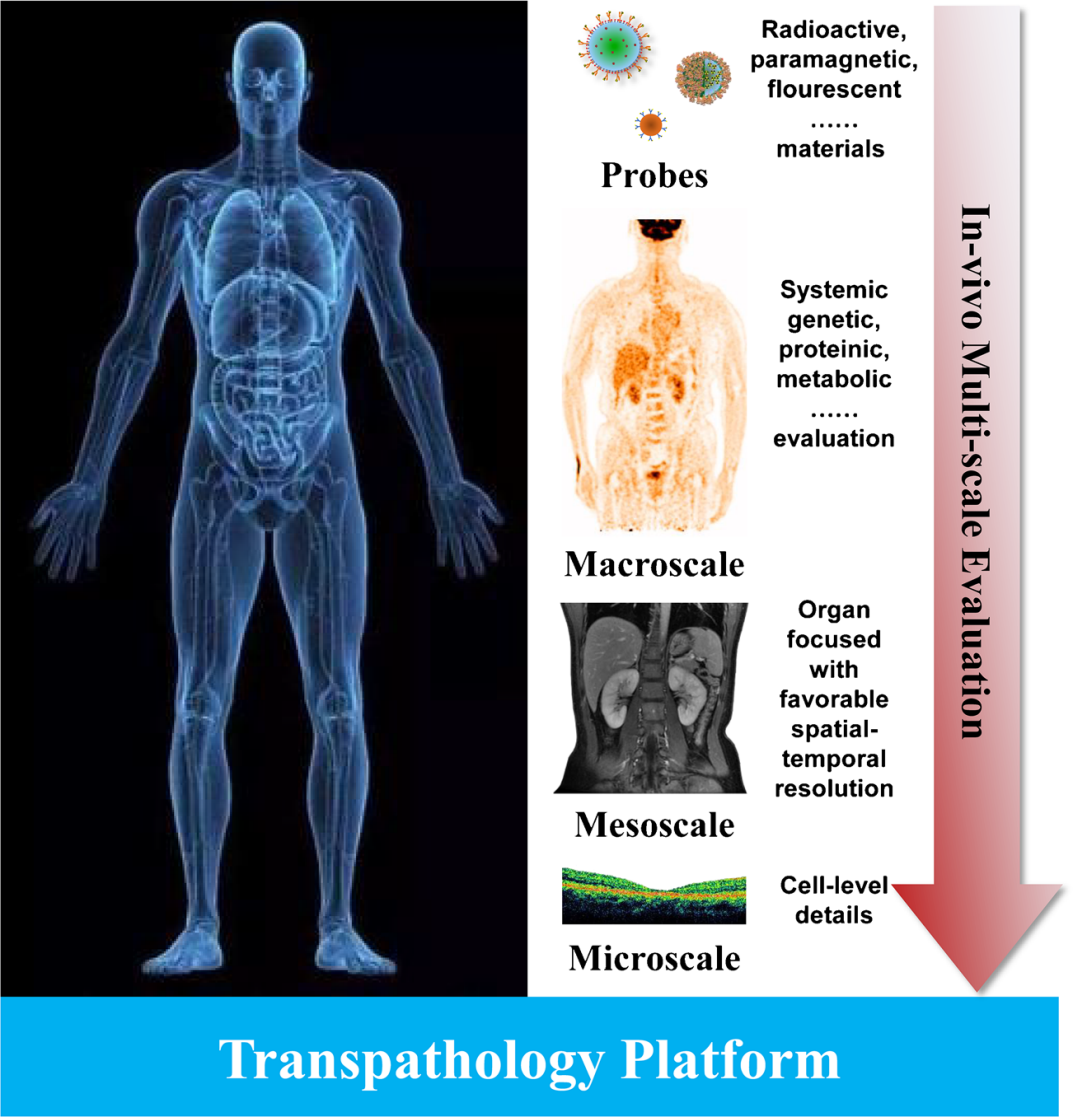
A schematic diagram of transpathology

As diseases are always accompanied by complex processes, an evaluation system for patients with multi-level and multi-perspective is essential to obtain and provide comprehensive pathophysiological information. In the system of transpathology, the multiscale imaging can be divided into three levels according to the spatial resolution: macroscale imaging approaches represented by PET/CT, mesoscale imaging approaches represented by MRI, and microscale imaging approaches represented by optical imaging. A schematic diagram of transpathology is shown in Fig. 1. And imaging modes commonly used in clinical practice are listed in Table 1.

**Table 1:** Classical imaging modalities of transpathology in clinical practice

Imaging technique	Source of imaging	Spatial resolution	Tissue penetration depth	Sensitivity	Examples for probes	Ref
Single-photon emission computed tomography (SPECT)	γ-ray	8–10 mm	No limit	pM	Radionuclides (^99m^Tc, ^201^Tl, ^111^In, ^131^I, ^123^I, ^67^Ga)	[56]
Positron emission tomography (PET)	Positron emitters	4–5 mm	No limit	pM	Radionuclides (^18^F, ^11^C, ^13^N, ^15^O, ^64^Cu, ^68^Ga)	[57]
Computed tomography (CT)	X-ray	1–2 mm	No limit	mM	High-atomic-number atoms (iodine, barium sulfate)	[58]
Magnetic resonance imaging (MRI)	Radiofrequency waves	0.5–1 mm	No limit	mM to μM	Para-(Gd^3+^) or superparamagnetic (Fe_3_O_4_) materials	[59]
Ultrasound (US)	Ultrasound waves	0.3–1.1 mm	Few centimeters	n.c.	Microbubbles	[60]
Optical coherence tomography (OCT)	Light waves	10–20 μm	Few millimeters	n.c.	Albumin microsphere, near-infrared dyes, gold nanoshells	[61]
Confocal microscopy (CM)	Light waves	0.5–1.25 μm	200–300 μm	n.c.	Aluminum chloride, indocyanine green, sodium fluorescein	[62]

*pM*, pmol/L; *mM*, mmol/L; *μM*, μmol/L; *n.c.*, not well characterized

### Macro techniques in diagnostic “transpathology”

Traditional pathology mainly focused on the localized specimens, while a macroscale (> 1 mm) evaluation of the lesion as well as patients at the whole-body level would provide a full understanding of the disease phenotype. Macroscale transpathology would undoubtedly lead to a better choice for therapy based on the overall profile of disease stage and biological features. PET/CT is the representative imaging mode for noninvasive macroscale evaluation.

PET is a representative technique of molecular imaging to evaluate the molecular, metabolic, and functional status of living organisms, based on the principle of radioactive tracing. As PET enables in vivo quantification of radioisotopes in nanomolar to picomolar range [63, 64], it can be used to assess protein binding, receptor availability, transporter systems, signal transduction, and gene expression [64–66]. With one administration of the radiotracer, accompanied by the agent distributed throughout the body, the whole body can be imaged at once, which greatly helps to better evaluate the overall condition of the patient. The deficiency of PET molecular imaging is the relatively lower spatial resolution, which is generally 4–5 mm by using routine clinical instruments in phantom studies, and the resolution realized in patients could be lower [67, 68]. By using dedicated scanners, the resolution limits could achieve 1–2 mm for micro-PET systems [69]. Recent developments of PET in geometric coverage, detector efficiency, and timing resolution may also provide significant gains in sensitivity [70]. Besides, to remedy such insufficiency, PET imaging has been extensively partnered with CT and MRI imaging. The emergence of PET/CT and PET/MRI has tremendously taken the advantage of radiology and molecular imaging, which greatly achieves the integration of imaging approaches at different scales in the same platform. These instruments sparked the studies involved in investigation of molecular information, such as tumor and neuropsychiatric diseases. For example, prostate-specific membrane antigen (PSMA) PET/CT are increasingly used to simultaneously evaluate both biochemical information and skeletal lesions in patients with prostate cancer [71]. And ^18^F-florbetaben or ^11^C-Pittsburgh Compound-B (^11^C-PIB) PET/MRI could offer the chance to obtain both amyloid pathology and neuronal injury biomarkers read-outs within one imaging session for patients with Alzheimer disease [72].

Despite the limited resolution, researchers have made great effort to relate PET data to microscopic images. By using X-ray film, phosphor imaging plates, beta imaging systems, or photo-nuclear emulsion, the technique of autoradiography has been widely used for decades to determine the localization of a radioactive substance, either introduced into a metabolic pathway, bound to a receptor or enzyme, or hybridized to a nucleic acid [73–75]. Various autoradiographic techniques, including whole-body autoradiography and the micro-autoradiography, could offer high-resolution images to determine the drug distribution at macro and micro level [75]. And by coupling with immunohistochemical staining methods, the co-localization of drug entities and cellular targets could be achieved [76]. Through the comparison with autoradiographic images, results of PET could be better verified and interpreted.

PET has played an increasingly important role in the diagnosis and treatment of diseases in various fields [77]. In the field of oncology, PET molecular imaging can now help assessing the proliferation level, protein expression, and gene mutations for local tumor lesions [78–81]. It is noted that these examinations were mostly performed in isolated tissues in the past and were impossible to evaluate the spatial heterogeneity of the tumor, nor to carry out long-term follow-up observation. In addition to local lesions, PET can evaluate the general condition of patients [82]. The tumor has been widely regarded as a systemic disease. The pattern assessment of local as well as whole body can better reveal the changes of local lesions and the difference in the systemic state of patients during tumor onset and progression. For some vulnerable organs, such as the brain and heart, pathological biopsy is difficult, and the lack of pathological specimens in these organs could affect the exploration of pathophysiological mechanisms for related diseases. In recent years, with the development of PET technology, the pathophysiological changes of many major neurological diseases, including Alzheimer’s disease, Parkinson’s disease, and psychiatric disorders, have been explored with the help of PET molecular imaging [83–85]. Similarly, for some heart diseases such as ischemic infarction, PET can sensitively detect the infarcts and evaluate the severity of the lesion, thus playing a key role in the diagnosis, evaluation, and treatment [86, 87].

In general, PET is a representative macroscale transpathological approach, which enables the visualization of localized molecular events and realizes whole-body dynamic imaging at macroscale, thus providing a comprehensive insight into the patient.

### Meso techniques in diagnostic “transpathology”

In addition to the macroscale evaluation, transpathological evaluation at mesoscale (500 μm–1 mm) would provide elaborate information of pathophysiological processes for diseased tissues and organs, thereby linking cellular (microscale) and whole-body (macroscale) events. MRI is the representative imaging mode for in vivo mesoscale evaluation.

MRI is a noninvasive imaging technique based on nuclear magnetic resonance of atoms within the body induced by the application of radio waves [88–90]. The different patterns of relaxation time for tissues would generate the variance in nuclear magnetic resonance signals [91]. Compared to other imaging modalities that are commonly used, such as CT and PET, MRI has the intrinsic advantage to provide multifaceted and excellent image contrast, especially for soft tissues, without utilizing ionizing radiation. Besides, MRI can be performed in any orientation without postprocessing image reconstruction. On the contrary, MRI has a limitation in imaging tissues with fewer hydrogen protons, and since most MRI schemes rely on acquiring one line of k-space at once, the scanning is relatively time-consuming.

The spatial resolution of MRI commonly used in clinical practice is about 1 mm. With the development of ultrahigh-field MRI technologies, the resolution of images is constantly improving, which reached 0.5 mm in a 7-T machine [59]. MRI has played an indispensable role in guiding the tumor resection, grading the infarction of brain and heart, and evaluating the inflammation [92–95]. By using multi-sequence scanning method, MRI can sensitively distinguish different tissue types based on the pattern of magnetic resonance signals. In addition, magnetic resonance spectroscopy (MRS) technology can utilize the magnetic resonance phenomena and chemical shift effects to analyze the chemical composition of the lesions, including creatine, choline, n-acetylaspartate, and lactic acid, which is useful for determining disease types and studying the dynamic pathological changes [93]. Another advantage of MRI is the high temporal resolution. The cardiac magnetic resonance (CMR) sequence is able to evaluate the cardiac structure and function in a state of beating, so as to better assess the wall motion abnormalities [96]. Blood-oxygenation-level-dependent (BOLD) imaging is another important technique used to generate images in functional MRI (fMRI) studies, relying on regional differences in cerebral blood flow to delineate regional activity [97]. This technology has greatly expanded our understanding of the functional connections for different brain regions, and provided important additional evidence for the isolated anatomical neural circuit experiments.

In summary, MRI is a representative mesoscale transpathological approach, performing high-resolution imaging of local organs with favorable spatial-temporal resolution, thus enabling the exploration of pathophysiological processes in a relatively detailed and holistic perspective.

### Micro techniques in diagnostic “transpathology”

The ever-developing techniques now also enable a noninvasive evaluation of disease in microscale (< 500 μm), which would provide pathophysiological events at cellular and molecular level. The integration of in vivo microscale information would greatly expand our understanding of disease mechanisms and reshape the pathological practice in the future. Optical imaging is the representative mode of transpathology in microscale.

Optical imaging instrumentation includes a large number of technologies ranging from aided visual inspection by magnifying glasses or video endoscopes to light microscopy, as well as techniques using computational algorithm to generate diagnostically relevant information. With its unique spatial resolution capabilities, optical imaging is able to provide subcellular details as well as mesoscopic structures of tissue and organs. Compared to MRI, CT, and PET, the advantages of optical imaging include lack of ionizing radiation exposure, high spatial resolution, and the feature of real-time imaging. The inadequacy of penetration depth due to tissue scattering and light absorption prevents its application for whole-body imaging. However, for intraoperative guidance, in which tumors can be visualized directly, this is a relatively minor disadvantage [98].

Given its lack in imaging depth, optical imaging is primarily used to study easily accessible outer surfaces of organs, such as the skin and the inner lumen of organs that can be reached by endoscopes or catheters, including the cardiovascular system, the gastrointestinal tract, oral cavity, larynx down to the lung, bladder and urethra, cervix, and uterus. The deepest noninvasive penetration is achieved through the eye down to the retina, which takes advantage of the natural transparency of the ocular media. Optical coherence tomography (OCT) is applied with great success in the eye as well as in inner organs through endoscopes [99]. Besides, confocal microscopy (CM) has been widely utilized in skin diseases, like melanoma and inflammatory, aiding not only an in vivo diagnosis of skin abnormalities but also choosing better therapies and following the patient’s response to treatment [62].

Despite the limitation of penetration depth, optical imaging is an imaging modality matching traditional pathological images on the scale. With details provided in cellular level, optical imaging would play an important role in providing dynamic interactions of cellular processes and relating in vivo imaging to microscopic data ex vivo. Besides, the increasing need to study biological processes in vivo also drove the development of novel photonic methods. Advances in technologies, including multiphoton microscopy, deep optical imaging using the second and third near-infrared spectral windows, and novel imaging methods like photoacoustic imaging, now enabled imaging of deeper tissues structure [7, 100]. The combined use of optical imaging with high-resolution anatomical methods may also offer more accurate images by improving the optical reconstruction algorithm [101].

Although there is still a long way to go for optical imaging to be applied in evaluating deep tissues in vivo to date, optical access to intact organs and even to some entire mammals ex vivo could be achieved with subcellular resolution by using tissue-clearing techniques, which equilibrate the refractive index of the sample to reduce inhomogeneities in light scatter [102]. The tissue-clearing technique is especially valuable to provide an unbiased exploration of biological information in the whole organism. In addition, tissue-clearing chemistry enables whole-organ antibody labelling, thus helps to provide the structural and functional information on complex mammalian bodies and large human specimens at cellular and subcellular level [103]. 

To sum up, optical imaging is a representative microscale transpathological approach, providing targeted molecular contrast and resolution which is unmatched by other modalities, and thus, holding great potential to obtain quantifiable details of complex biological mechanisms in live and intact tissues.

## Multimodal probe technology to make the whole body transparent

Probes should be the key to visualize the complicated pathophysiological progresses. However, each of the existing methods has its limitations, and it is difficult to meet the requirements of sensitivity, specificity, and targeting simultaneously. The multimodal molecular probe can carry out multi-mode imaging at the same time, which overcomes the shortage of single imaging mode, realizes the superiority complementation, and widens the application range of molecular imaging technology.

### PET/optical imaging bimodal probe

The optical imaging has a high signal-to-noise ratio and good temporal-spatial resolution, but with insufficient penetration depth, and lacks the quantification ability. In contrast, PET has favorable tissue penetration depth, sensitivity, and quantitative effects, but with low spatial resolution, and is difficult to be used in intraoperative applications. The combination of PET and optical imaging would offer complementary clinical applications, enabling the noninvasive preoperative whole-body imaging and identification of tumor margins during surgery, respectively. The commonly used PET/optical bimodal imaging probes are based on the combination of radionuclides and fluorescent dyes, including ^64^Cu quantum dots [104], ^86^Y near-infrared materials [105], or ^89^Zr near-infrared [106].

### MRI/optical imaging bimodal probe

MRI has the characteristics of high soft tissue resolution, enabling multi-directional and multi-parameter imaging, but its sensitivity and targeting are relatively low. MRI/optical imaging bimodal probe is a relatively mature technology, with high resolution of MRI and high sensitivity of optical imaging. The combination of the two imaging methods improves the diagnostic accuracy. With the development of nanotechnology, more and more materials are used to construct MRI/optical probes that present paramagnetic and fluorescence properties simultaneously, such as fluorescent dye, functional quantum dots, nano gold, and rare earth materials [107].

### PET/MRI imaging bimodal probe

Currently, PET/MRI imaging has been used in routine clinical practice. Due to its advantages of the sensitivity, specificity, and resolution over stand-alone PET and MRI, PET/MRI has got increasing attention in clinical practice in many areas such as neurodegenerative diseases. All agents used for PET/CT are also applicable for PET/MRI. Additionally, increasing novel bimodal molecular probes are gradually used in scientific research and clinical practice for PET/MRI, such as Gd^3+^-containing contrast agents with radioligands [107], ^124^I nanomaterials [108], and ^64^Cu nanomaterials [109].

### PET/MRI/optical imaging trimodal probe

In recent years, three-modal probes have gradually become a research hotspot. A recent study has reported PET/MRI nanosilicon probes with enhanced near-infrared fluorescence (NIRF) signals to realize the monitoring of sentinel lymph nodes in vivo [110]. And another study demonstrated PET–NIRF–MRI trimodality nano-particles for glioblastoma tumor imaging, showing massive accumulation in lesions, high extravasation rate, and low uptake of the particles by macrophages at the tumor area [111]. However, more probes are needed to better support the trimodal molecular imaging.

## Multiscale integration and fusion platform of “transpathology”

The imaging techniques mentioned above can be utilized to visualize the pathophysiological processes in various perspectives. However, it is essential to integrate all the methods and render the complex information in multiscale comprehensively. In recent years, complex AI technologies have been greatly developed with the emergence of deep learning algorithms, the exponential growth of computing power, and the abundant big data resources, as well as the computer’s brain-like capabilities. AI has empowered medical industry to make amazing achievements in virtual physician assistant, medical record, drug research, gene sequencing, and image-assisted diagnosis, etc. Among them, the combination of medical image and AI is the most promising field.

Data is still the core and key component for developing AI-based displaying and analytic algorithms. Picture Archiving and Communication System (PACS) has been widely used for storing and transmitting digital images in hospitals, showing the great potential to integrate comprehensive medical information in various hospital systems. However, these data are rarely sorted out in label, annotation, separation, and quality assurance. The management of medical data needs trained professionals, which is very expensive in time and cost. It has become a major bottleneck in the development of AI model for automated clinical solutions [112]. Standardized data, especially the data of multiple imaging modes and anatomical positions, is particularly important in the field of medicine [113].

The significance of diagnostic information registration and fusion lies in the integration of the advantages of diagnostic information in different modes and different times, such as spatial-temporal information, functional-structure information, or global-local information. Representative studies include the fusion of intraoperative fluorescence in preoperative MRI data to improve the ability of tumor region recognition [114]; the method of intraoperative MRI non-rigid registration to calculate brain drift [115]; and the rapid fusion of intraoperative ultrasound and multi frame endoscopic images to present 3D ultrasound surface texture information [116].

In the diagnosis and treatment of complex structural areas, the presentation of diagnostic information is of great importance. The data obtained directly from the diagnostic device is usually two-dimensional image or signal. Three-dimensional volume rendering and surface rendering are the basic visualization methods for volume data. Before endoscopy and interventional treatment, the virtual operation scene and route feedback based on the preoperative diagnosis data will help doctors to accurately judge the pipeline bifurcation and reach the focus position for treatment faster [117, 118]. And with the progress of image segmentation methods and the popularity of computer graphics processors, it has been able to automatically render large-scale volume data in real-time visualization [119]. In addition, more and more open-source software and toolkits have integrated common visualization algorithms well, greatly facilitating the development process for researchers [120].

Besides, increasing multiscale imaging systems are emerging. By using single platform to achieve multi-level imaging, image registration could be eased. A recent study described a multiscale imaging platform that is sensitive to differences in tissue structure ranging from the organ macroscale to the subcellular nanoscale [121]. Five modalities were incorporated onto the integrated platform: ultrasound, second harmonic generation microscopy, multiphoton microscopy, optical coherence tomography, and enhanced backscattering. As the sample was imaged in the same condition for each modality, the task of comparing structures between modalities was greatly simplified. Integrated multiscale imaging systems are helpful to relate images from different scales, and facilitate the in vivo pathological evaluation. Additionally, internal fiducials like blood vessels and airway tree structures, as well as external fiducials like a sample holder, have also been used to facilitate the image co-registration [122–125]. The emerging multiscale imaging systems are summarized in Table 2.

**Table 2:** Emerging multiscale imaging systems

Imaging modalities	Target	Co-registration strategy	Main finding	Ref
US, SHGM, MPM, OCT, and EBS	A rabbit eye	Five modalities were incorporated onto a single platform and imaging tissue samples in the same condition.	A multiscale imaging platform was developed and this system simplified the task of comparing like structures between modalities.	[121]
MRI, μMRI, μCT	Angiogenesis in a mouse breast cancer model	The resolution gap between ex vivo μCT and in vivo MRI was bridged using intermediate resolution ex vivo μMRI.	An integrated platform was developed for characterizing angiogenesis at multiple spatial scales in a human breast cancer model.	[126]
SEM, μCT, SRμCT	Brain vasculature in mouse	A specialized sample holder was used to facilitate registration of images from different modalities.	This system was able to reveal whole brain microvascular features with unprecedented resolution (~1 μm).	[123]
MRI, CT, MPM	Microvascular in a breast cancer model	Internal vascular fiducials were employed to facilitate image integration.	An elastic multiscale image co-registration method (VASFID) was developed.	[125]
In vivo MRI, ex vivo MRI, and histology	Lung inflammation in a diseased mouse	Airway tree structures in histology were compared with ex vivo MRI to facilitate co-registration.	A use case was presented to evaluate the co-registration framework in the context of studying chronic inflammation in a diseased mouse.	[124]
μCT, FIB-nt, SEM, STEM	Sediment flocs	Following resin embedding, fiducial markers were implanted in the base of each resin block to facilitate data co-registration.	The integration of multiscale techniques generated new understanding of floc composition, and this strategy could also be used in biomedicine.	[122]

*US*, ultrasound; *SHGM*, second harmonic generation microscopy; *MPM*, multiphoton microscopy; *OCT*, optical coherence tomography; *EBS*, enhanced backscattering; *SEM*, scanning electron microscopy; *μMRI*, ex vivo MR microscopy; *μCT*, microcomputed tomography; *SRμCT*, synchrotron radiation microcomputed tomography; *FIB-nt*, 3D focused ion beam nanotomography; *SEM*, scanning electron microscopy; *STEM*, scanning transmission electron microscopy

## Future perspectives

The development of molecular imaging-based transpathology will be an important content in the biomedical era. Currently, anatomy, histology, and cellular pathology are undergoing the re-layout and transformation of disciplines to meet the establishment of precision pathology. Molecular imaging would play a key role in bridging the multiscale pathological changes. With the continuous progress made in advanced clinical multimodal imaging diagnosis, major technological breakthroughs ranging from macroscopic approaches to microscopic techniques are emerging, and the transformation from basic scientific research to clinical medicine is getting increasingly mature. With the development of genomics, proteomics, and metabonomics, the organic combination of macro and micro imaging technology, and the progress in information and AI technology, clinical pathology will be promoted towards the pattern of cross-scale, multi-mode “transparent pathology.”

The comprehensive multiscale diagnosis system based on “transpathology” combined with clinical information will make a timely and accurate diagnosis for patients, and guide clinicians to make optimal and individualized therapeutic regimes. The establishment of an integrated diagnosis model with “transpathology” would help clinicians to conduct pathological practice beyond the current scope of pathology. The pattern of multiscale transpathological practice, which comprehensively visualizes the ongoing events in the patient by using an integrated scan, would further drive current medicine to precision medicine in the future.
